# Factors limiting sulfolane biodegradation in contaminated subarctic aquifer substrate

**DOI:** 10.1371/journal.pone.0181462

**Published:** 2017-07-20

**Authors:** Christopher P. Kasanke, Mary Beth Leigh

**Affiliations:** Institute of Arctic Biology, University of Alaska Fairbanks, Fairbanks, Alaska, United States of America; VIT University, INDIA

## Abstract

Sulfolane, a water-soluble organosulfur compound, is used industrially worldwide and is associated with one of the largest contaminated groundwater plumes in the state of Alaska. Despite being widely used, little is understood about the degradation of sulfolane in the environment, especially in cold regions. We conducted aerobic and anaerobic microcosm studies to assess the biological and abiotic sulfolane degradation potential of contaminated subarctic aquifer groundwater and sediment from Interior Alaska. We also investigated the impacts of nutrient limitations and hydrocarbon co-contamination on sulfolane degradation. We found that sulfolane underwent biodegradation aerobically but not anaerobically under nitrate, sulfate, or iron-reducing conditions. No abiotic degradation activity was detectable under either oxic or anoxic conditions. Nutrient addition stimulated sulfolane biodegradation in sediment slurries at high sulfolane concentrations (100 mg L^-1^), but not at low sulfolane concentrations (500 μg L^-1^), and nutrient amendments were necessary to stimulate sulfolane biodegradation in incubations containing groundwater only. Hydrocarbon co-contamination retarded aerobic sulfolane biodegradation rates by ~30%. Our study is the first to investigate the sulfolane biodegradation potential of subarctic aquifer substrate and identifies several important factors limiting biodegradation rates. We concluded that oxygen is an important factor limiting natural attenuation of this sulfolane plume, and that nutrient amendments are unlikely to accelerate biodegradation within in the plume, although they may biostimulate degradation in *ex situ* groundwater treatment applications. Future work should be directed at elucidating the identity of indigenous sulfolane-degrading microorganisms and determining their distribution and potential activity in the environment.

## Introduction

Anthropogenic organic compounds are present as environmental contaminants throughout the world [1]. Many of these chemicals were engineered for industrial purposes, in which resistance to degradation is advantageous [2]. However, this desirable characteristic becomes problematic when compounds of this nature are released into the environment since recalcitrance correlates to persistence [3]. In addition, many synthetic organic compounds are designed for specific applications, which creates a diverse suite of potential environmental contaminants that are unique in their reactivity and persistence [4]. Often these compounds are not included in routine environmental monitoring protocols, as they are not regulated or well researched in terms of their toxicity or fate in the environment. This lack of understanding is a cause for concern when an unregulated industrial solvent enters a residential drinking water source. An example of this scenario occurred in Interior Alaska, where accidental industrial releases of sulfolane (tetrahydrothiophene 1,1 dioxide) from a petroleum refinery created one of the largest groundwater contamination plumes in the state (Alaska Department of Environmental Conservation, personal communication).

Sulfolane is an anthropogenic organosulfur compound used in various industrial processes, such as natural gas and petroleum refining, with 18,000–36,000 tons produced worldwide annually [5]. Sulfolane is miscible in water, has a low affinity for aquifer materials (K_d_ = 0.008–0.14), and is more stable than many common co-contaminants such as hydrocarbons and diisopropylamine [6,7]. These qualities make sulfolane a mobile and persistent groundwater contaminant once released into the environment [8]. Although the human health effects are unknown, toxicity studies, in which rats were exposed to sulfolane through their drinking water, found lowered white blood cell counts in females and neuropathy in males after 90 days [9]. No other studies have reported the effects of chronic, low-dose sulfolane exposures on humans or other animals [10].

There are no practical strategies to actively remediate such a large sulfolane plume in this region and remediation efforts have been recently replaced with groundwater monitoring [11]. However, previous research has demonstrated that sulfolane can be biodegraded by microorganisms found in sludge from wastewater treatment plants, biologically activated carbon, and in aquifer materials [12–14]. Exploiting the metabolic capabilities of microorganisms naturally occurring in areas of contamination, using techniques such as monitored natural attenuation or biostimulation, may be a way to remediate sulfolane-contaminated aquifers. Before employing bioremediation strategies, an understanding of the sulfolane biodegradation potential of microorganisms present in contaminated environments and the environmental factors controlling their activity must be achieved. Prior to this study no such information existed for subarctic aquifers.

The ability of indigenous microorganisms from a contaminated aquifer to perform sulfolane biodegradation has been reported previously in western Canada [13,15,16], and Australia [17]. Aerobic incubations using aquifer sediment from western Canada revealed that lower temperatures (i.e. 8°C vs. 28°C) limited sulfolane biodegradation and that the addition of nitrogen and phosphate stimulated biodegradation rates [13,15,18]. The biochemical pathway for sulfolane biodegradation has not yet been elucidated, but sulfate, one predicted end product of sulfolane biodegradation, was produced as sulfolane degraded [19]. Anaerobic sulfolane biodegradation studies that have been reported in the scientific literature have not generated consistent results. One study suggests sulfolane is readily degradable under unspecified anaerobic conditions [17], while another found inconsistent anaerobic biodegradation only under nitrate- and Mn(IV)-reducing conditions [15]. This discrepancy may be due to the difference in experimental methods and the biogeographic differences in microbial communities associated with the substrates tested (i.e. Australian and Canadian aquifer materials respectively) [20]. Subarctic aquifers are generally cold and nutrient poor; conditions that are known to limit microbial activity. Therefore, it was necessary to assess the sulfolane biodegradation potential of the microbial community associated with subarctic aquifer substrate.

We conducted microcosm studies to assess the microbial (aerobic and anaerobic) and abiotic degradation potential in subarctic aquifer substrates from a contaminated groundwater plume in the Interior Alaska city of North Pole, Alaska. Our objectives were to identify degradative processes that contribute to the fate of sulfolane in the environment, and to identify environmental factors that may limit them *in situ*. Groundwater and sediment were combined as the inoculum in the majority of incubations since a greater portion of aquifer microbiota are thought to be associated with aquifer sediment [21]. Because the water table in North Pole, Alaska, is shallow and groundwater pumping followed by storage or treatment is often required for construction activities, we also conducted a comparative study to determine the biodegradation potential of the planktonic microbial community associated with the groundwater alone. We assessed the potential stimulatory effects of nutrients, including mineral nutrients and a complex organic amendment (beer fermentation settlings) on biodegradation rates in order to evaluate nutritional limitations and to possibly identify biostimulation strategies. Since hydrocarbon contamination (primarily jet fuel) co-exists with sulfolane in portions of the North Pole aquifer [22], we also examined the impact of aliphatic hydrocarbons on sulfolane biodegradation rates. Sterile microcosms were also run in parallel and in the dark to assess abiotic chemical degradation processes. We hypothesized that sulfolane degradation in subarctic aquifer substrate occurs primarily as the result of microbial processes, and that biodegradation is limited by oxygen and *in situ* nutrient availability.

## Materials and methods

### Aerobic microcosm studies

The North Pole, Alaska, aquifer is part of the greater Tanana River aquifer, which is fed by the Alaska Range. Subsurface samples used as inoculum for aerobic microcosm studies were collected from Flint Hills Resources property located in North Pole, Alaska (64.7511° N, 147.3519° W) with permission of the property owners. Sulfolane use at this site began in 1985 and ended in 2014 when the plant stopped refining crude oil. The plume morphology and fate is impacted by the presence of discontinuous permafrost in the aquifer and groundwater sulfolane levels range from 0–34.8 mg L^-1^ [22]. All sediment used in this study was collected in March 2013, from one sampling event of augured material from the installation of a new monitoring well at depths between 3 and 9 m below ground surface. Sediment was stored at 4°C (up to 13 months) and sieved through a 2 mm screen prior to use. Twenty liters of groundwater was collected in September 2012 using a peristaltic pump and stored at 4°C until use (up to 18 months). Groundwater came from an existing monitoring well approximately 30 m from where sediment was collected. The well was screened 18.25 m below the ground surface and has stable historical sulfolane concentrations of approximately 125 μg L^-1^ [22]. The top of the water table at time of sampling was 3 m below ground surface and the aquifer has an average temperature of 3.4°C [22].

#### Incubations of aerobic sediment-groundwater slurries ± mineral nutrients

Aerobic sulfolane degradation rates were assessed at two different sulfolane concentrations. “High concentration” slurries contained 25 g of aquifer sediment, 100 ml of groundwater, and sulfolane to a target concentration of 100 mg L^-1^, and “low concentration” slurries contained 50 g of aquifer sediment, 250 ml of groundwater, and sulfolane to a target concentration of 500 μg L^-1^ including background contamination. To observe the effects of nutrient amendment on biodegradation rates at high and low sulfolane concentrations, a Bushnell-Hass (BH) mineral nutrient solution was added to a subset of both slurry types (5 replicates). Each BH-amendment added 8 μg L^-1^ magnesium sulfate, 1.8 μg L^-1^ calcium chloride, 90 μg L^-1^ monopotassium phosphate, 90 μg L^-1^ dipotassium phosphate, 90 μg L^-1^ ammonium nitrate, and 4.5 μg L^-1^ ferric chloride. Two types of experimental controls were established: no-sulfolane controls and sterile controls (3 replicates) (Table 1). No-sulfolane controls were created exactly as described above, but without sulfolane addition. Sterile controls were autoclaved. Sterile aerobic conditions were maintained by loosely covering all incubation vessels with aluminum foil and shaking at 100 rpm at 4°C, that temperature being the approximate year-round average of the North Pole aquifer [22]. Aliquots of liquid (1–2 ml) were routinely sampled every 5–7 days for sulfolane and sulfate analysis. High concentration incubations were monitored for 106 days, at which point monitoring ceased due to logistical reasons. Low concentration incubations were monitored for 47 days; more time than was necessary to no longer detected sulfolane in the live slurries.

**Table 1:** Experimental design for aerobic sulfolane biodegradation microcosm studies.

	Treatment groups	Replicates	Sulfolane	Microbes	Other amendment
**High and Low Concentration Slurries**	Live slurry	5	+	+	None
	Live slurry (N)	5	+	+	Mineral Nutrients*
	Sterile control	3	+	-	None
	Sterile control (N)	3	+	-	Mineral Nutrients*
	No sulfolane control	3	-	+	None
	No sulfolane control (N)	**3**	-	+	Mineral Nutrients*
**Hydrocarbon Co-contaminat Slurries**	Live slurry	5	+	+	None
	Live slurry (K)	5	+	+	Kerosene
	Sterile control	3	+	-	None
	Sterile control (K)	3	+	-	Kerosene
	No sulfolane control	5	-	+	None
**Groundwater Only**	Live slurry	3	+	+	None
	Live slurry (N)	3	+	+	Mineral Nutrients*
	Live slurry (O)	3	+	+	Organic Nutrient**
	Sterile control	3	+	-	None
	Sterile control (N)	3	+	-	Mineral Nutrients*
	Sterile control (O)	3	+	-	Organic Nutrient**

Conditions tested were high (100 mg L^-1^) and low sulfolane concentrations (500 μg L^-1^) in sediment slurries, hydrocarbon and sulfolane co-contamination in sediment slurries, and biodegradation in groundwater only. (N) indicates treatments amended with mineral nutrients. (K) indicates kerosene amendment. (O) indicates amendment with organic nutrients.

* Amended with an 11-fold dilution of a 1X Bushnell-Haas mineral nutrient broth.

** Amended with a complex organic nutrient solution. In the high concentration incubations there were only two replicates of the nutrient amended sterile control while there were three replicates in the low concentration incubations (refer to the results section for a detailed explanation).

#### Aerobic incubations using groundwater only

It has been suggested that the majority of aquifer bacteria are attached to sediment particles rather than living as planktonic cells in the groundwater [23]. The biodegradation potential of sulfolane by planktonic microbes residing in groundwater alone was examined in microcosms similar to those described above, but with the omission of aquifer sediment. Groundwater-only microcosms were created by combining 150 ml of groundwater and sulfolane to a target concentration of 500 μg L^-1^ including background contamination. The flasks were then divided into different treatment groups (Table 1). The effect of mineral nutrient and complex organic nutrient amendments was assessed separately. Microcosms amended with mineral nutrients contained BH mineral nutrient broth as described above and obtained by dilution. The complex organic nutrient solution used as an alternative amendment was created by autoclaving a four-fold dilution of fermentation settlings obtained from a local brewery. Microcosms amended with organic nutrients received 0.5 ml of the complex organic nutrient solution. Sterile controls were created by autoclaving a subset of each treatment group. Groundwater-only incubations were monitored for 80 days when sulfolane was no longer detectable in the nutrient amended treatment groups. All treatment groups were replicated in triplicate. Sampling conditions were the same as described above (Table 1).

#### Microcosms co-contaminated with sulfolane and hydrocarbons

Since petroleum hydrocarbons and sulfolane are both found in portions of the aquifer within the refinery property, co-contamination studies were conducted to assess the impacts of hydrocarbon co-contamination on sulfolane biodegradation. Sediment slurries were created using 25 g of aquifer sediment, 100 ml of groundwater, and sulfolane to a target concentration of 750 μg L^-1^ including background contamination. Kerosene was selected as a surrogate for jet fuel and diesel fuel, which are the primary forms of petroleum contamination onsite, since the mixtures are composed of a similar array of hydrocarbons (primarily aliphatic). Fifty microliters of kerosene were added to a subset of the microcosms (5 replicates) after sterilization through a 0.22 μm filter. Although kerosene is not miscible with water, constant agitation on a shaker table ensured that it was uniformly mixed in the amended microcosms. Controls, incubation conditions, and sampling were the same as previously described (Table 1). Sulfolane concentration in co-contaminant incubations was monitored for 22 days, at which point sulfolane was no longer detectable in the live slurries.

### Anaerobic microcosms

Aquifer sediment used as inoculum in anaerobic microcosm studies was obtained from a capped soil core from a depth of 5.25–5.75 m below ground surface, collected from the refinery property described above. After collection, samples were placed in gas-tight containers equipped with septa, flushed with N_2_ to maintain an anaerobic environment, and stored at 4°C until use approximately 2 months after collection. Groundwater used in these incubations was collected from a pre-existing monitoring well on refinery property that had historical sulfolane concentrations of approximately 500 μg L^-1^. Media bottles were filled to the top to eliminate oxygen in the headspace and stored at 4°C overnight to allow biological consumption of dissolved oxygen. Resazurin was added to a final concentration of 1 mg L^-1^. The groundwater was then degassed with N_2_ and reduced using sodium sulfide for the nitrate- and sulfate-reducing incubations. No reducing agent was added to the substrate used in iron-reducing microcosms.

#### Nitrate- and sulfate-reducing incubations

To evaluate sulfolane biodegradation potential in anaerobic aquifers, sulfolane biodegradation test microcosms were established under anaerobic conditions. For each anaerobic microcosm, 50 g of sediment was combined with 75 ml of groundwater. Microcosms were divided into two groups for nitrate-reducing and sulfate-reducing incubations. In the nitrate-reducing microcosms, KNO_3_ was added to a final concentration of 10 mM. In sulfate-reducing microcosms, Na_2_SO_4_ was added to a final concentration of 10 mM. There were 14 microcosm replicates of each reducing condition, which were divided into four treatment groups. Three microcosms were not amended and served as controls for background metabolic activity. To generate positive controls to verify the presence of an active microbial community, a relatively labile carbon source, benzoate was added to three microcosms to a final concentration of 50 mg L^-1^ molecular carbon. The remaining eight microcosms were amended with sulfolane to a final concentration of 50 mg L^-1^ molecular carbon, three of which were autoclaved as sterile biological controls and the other five were replicates to assess sulfolane biodegradation. All microcosms were incubated at 4°C in the dark and were not disturbed until sampling, which occurred eight times in 1021 days. Aliquots from all microcosms were periodically taken for sulfolane and sulfate/nitrate analysis. All activity was conducted under strict anaerobic conditions. Nitrate- and sulfate-reducing incubations were monitored for 1021 days.

#### Iron-reducing incubations

Twelve iron-reducing microcosms were established by combining 50 g of aquifer sediment and 65 ml of groundwater. Amorphous iron oxide was made in house, checked by X-ray diffraction to confirm amorphous structure, and added to each microcosm resulting in a final concentration of 37 mM per microcosm [24]. Three of the twelve microcosms received no further amendment in order to monitor background metabolic activity. Three others were amended with benzoate (final concentration 50 mg L^-1^ molecular carbon) and served as positive controls. The remaining six microcosms were amended with sulfolane to a final concentration of 50 mg L^-1^ molecular carbon; three served as the treatment group and three were autoclaved and used as sterile controls. All microcosms were incubated at 4°C in the dark and were not disturbed until sampling, which occurred four times in 391 days.

### Chemical analyses

#### Sulfolane extraction and quantification

To quantify changes in sulfolane concentration over time and among treatment groups, three rinses of dichloromethane were used to perform an organic liquid—liquid extraction of aqueous aliquots from each microcosm. An aqueous solution of sulfolane-d8 was added to monitor extraction efficiency. Nitrobenzene—d8 was used as the internal standard. All sulfolane quantification was done on an Agilent 5975 gas chromatography mass spectrometer (GC-MS) (Santa Clara, California). A fluorinated 30-m RTX– 200 column (Restek) was used for these samples as it separated based on lone pair electrons, allowing for exclusion of potential hydrocarbon co-contaminants. Two GC-MS methods were developed to analyze sulfolane content in both high (100 mg L^-1^) and low (500 μg L^-1^) sulfolane concentration incubations. High concentration incubation samples were analyzed with a method using splitless injection. Low concentration samples were analyzed using a pulsed-splitless injection method where the injection pressure was increased to 40 psi and held for one minute. The lowest quantifiable amount of sulfolane detectable in water samples was 40 μg L^-1^.

#### Nitrate, sulfate, and Fe(II) quantification

Nitrate and sulfate concentrations were analyzed using standard ion chromatography on a Dionex-200 liquid chromatograph coupled to a conductivity detector. A carbonate—bicarbonate buffer was used as the mobile phase. This instrument was demonstrated to be sensitive down to 1 mg L^-1^ for each ion of interest. Dissolved iron (Fe(II)) was measured using the Ferrozine assay [25] and was demonstrated to be sensitive down to 10 μg L^-1^.

### Statistical analyses

To determine if sulfolane degradation had occurred, treatment groups within the same experiment (Table 1) were compared to each other at each time-point using analysis of variance (ANOVA); sulfolane concentration being dependent on treatment type. A p-value of 0.05 or less was considered statistically significant. Due to the dramatic differences between treatment groups and low number of ANOVA tests performed, corrections for repeated measurements were not necessary. If significant differences in sulfolane concentrations were detected based on treatment (e.g. live slurry, sterile control) specific differences between treatments were identified using post-hoc Tukey tests. All values are reported ± standard deviation from the mean. All statistical analyses were run using R statistical software [26].

## Results

### Aerobic microcosms

#### Biological vs. abiotic degradation

**Fig 1:**
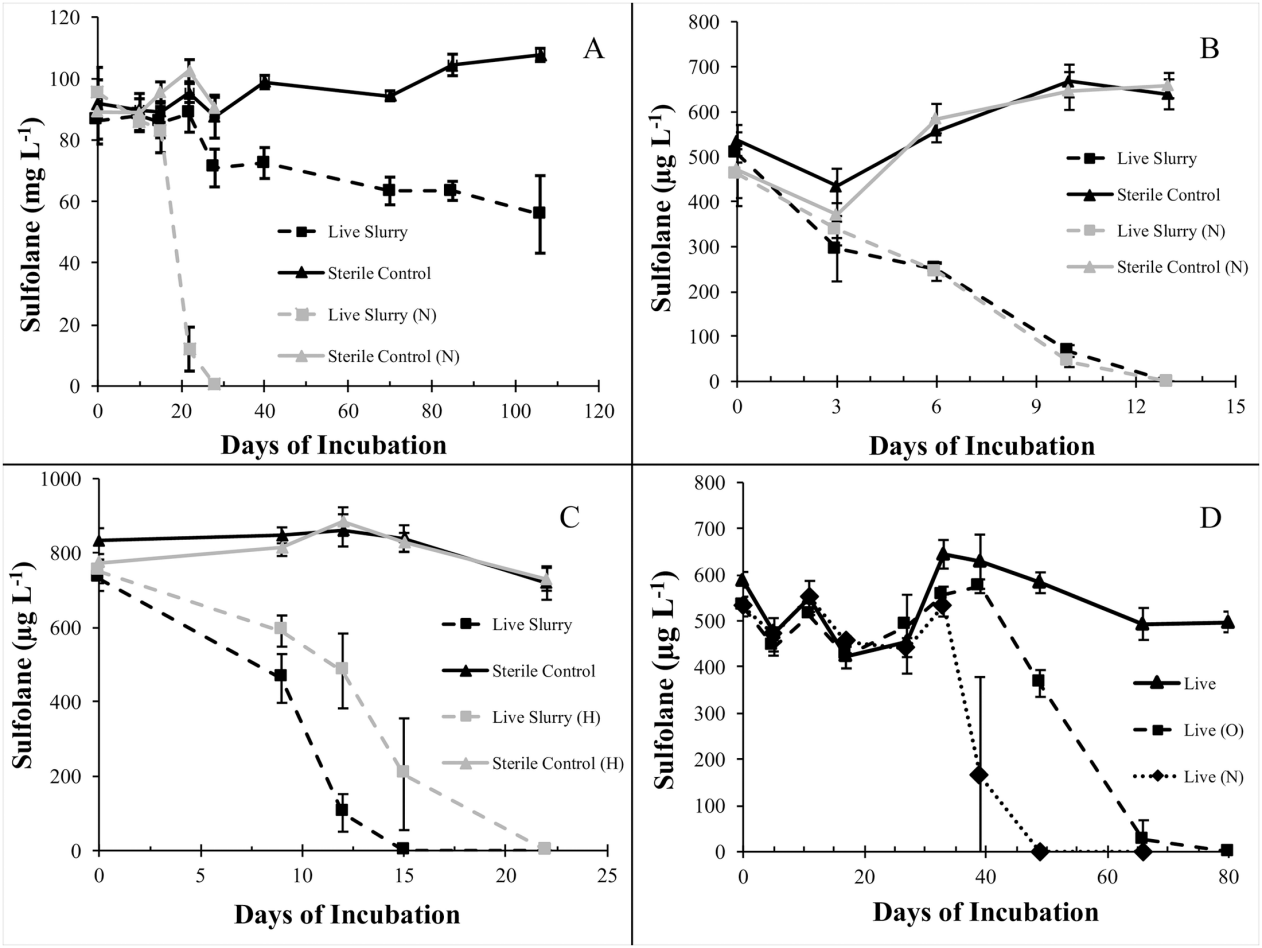
Sulfolane concentration over time in aerobic microcosm incubations. (A) Sulfolane biodegradation is nutrient limited in high concentration sediment slurry microcosms. (B) Sulfolane biodegradation is not nutrient limited in low concentration sediment slurry microcosms. (C)Hydrocarbon co-contamination retards the rate of sulfolane biodegradation in sediment slurry microcosms. (D) Nutrient amendment is necessary to stimulate sulfolane biodegradation in groundwater only microcosms. Live slurries contained an active microbial community and sulfolane. Sterile controls were heat-killed. (N) indicates amendment with a dilute mineral nutrient solution. (H) indicates treatments amended with hydrocarbons. (O) indicates treatments amended with a complex organic nutrient solution. Error bars indicate standard deviation from the mean.

Biodegradation was the only mechanism of sulfolane removal observed in aerobic microcosms (Fig 1) with no statistically significant losses being detected in sterile controls. One nutrient-amended sterile control replicate from the high concentration microcosm study was excluded from analysis, as sulfolane loss due to microbial contamination was identified after 15 days of incubation. In order to rule out abiotic degradation, a 59-day follow-up incubation of six sterile control replicates under the exact same conditions (i.e. 100 mg L^-1^ sulfolane amended with mineral nutrients) was performed. No sulfolane loss was observed in the sterile replicates, confirming biodegradation as the only mechanism of sulfolane loss in these incubations.

#### Effect of nutrient amendments at high and low sulfolane concentrations

The addition of a dilute mineral nutrient solution significantly increased the rate of sulfolane biodegradation in high concentration sediment-slurry microcosms (Fig 1A). For the first 10 days of incubation, there was no significant change in sulfolane concentration in any high concentration treatment groups. After 22 days of incubation, however, differences in sulfolane concentrations among treatments were detected (ANOVA, F_3,10_ = 195, p <0.001). By day 22, sulfolane concentrations in nutrient-amended live slurries had dropped from the initial level of 95.45 ± 8.18 mg L^-1^ to 12.02 ± 7.14 mg L^-1^, resulting in a significant concentration difference when compared to the sterile control (p <0.001). After 28 days of incubation, the mean sulfolane concentration in nutrient-amended slurries was below 1 mg L^-1^ (0.47 ± 0.7) with three of five replicates having no detectable sulfolane remaining. The unamended live slurries also contained less sulfolane than their sterile counterparts on day 28 (p = 0.028), and within 106 days of incubation, sulfolane concentrations had decreased from 86.45 ± 6.17 mg L^-1^ to 55.82 ± 12.61 mg L^-1^. The highest biodegradation rate observed in high concentration, unamended incubations was 2.93 mg L^-1^ day^-1^, while that in the nutrient-amended slurries was 6.19 mg L^-1^ day^-1^ (Fig 1A).

In contrast to the high-concentration sediment slurry microcosms incubated under aerobic conditions, the addition of a dilute mineral nutrient solution had no effect on the rate of sulfolane biodegradation at low sulfolane concentrations (p = 0.97) (Fig 1B). At 7 days of incubation, there were differences detected between the sterile controls and live slurries (ANOVA, F_3,12_ = 275, p <0.001) with the live treatment groups having significantly lower sulfolane levels than the sterile controls (p <0.001). By day 13, the sulfolane concentration dropped from 462.07 ± 54.41 μg L^-1^ and 506.53 ± 19.75 μg L^-1^ to below detection limits in all replicates of the nutrient amended and un-amended microcosms respectively. Sulfolane biodegradation occurred at an average rate of 38.96 μg L^-1^ day ^-1^. There was no lag time detected in biodegradation activity in live slurries, and no loss of sulfolane was observed in the sterile controls over the course of the 47-day incubation.

#### Effect of mineral and organic nutrients on sulfolane biodegradation in groundwater

Sulfolane biodegradation did not occur in groundwater-only microcosms without nutrient amendment during the 80-day incubation period (Fig 1D). Sulfolane biodegradation occurred more quickly in the live microcosms amended with mineral nutrients than in those amended with complex organic nutrients. ANOVA testing revealed differences in sulfolane concentrations due to treatment after 39 days of incubation (ANOVA, F_7,16_ = 10.83, p <0.001) attributed to sulfolane loss in the mineral nutrient treatment (p <0.001). Sulfolane loss was observed in microcosms amended with organic nutrients when compared to the sterile controls after 49 days of incubation (ANOVA, F_7,15_ = 32.86, p <0.001; post-hoc Tukey test, p <0.001). At that time (day 49) there was no detectable sulfolane remaining in the mineral nutrient treatment. After 80 days of incubation, sulfolane was no longer detected in microcosms amended with complex nutrients. Biodegradation rates were calculated to be 33.3 μg L^-1^ day^-1^ and 14 μg L^-1^ day^-1^ in the mineral and complex nutrient amended microcosms respectively. No sulfolane loss was observed in any of the sterile controls.

#### Effect of hydrocarbon co-contamination on sulfolane biodegradation

Sulfolane degraded more slowly in the presence of petroleum hydrocarbons when compared to the sulfolane-only microcosms (Fig 1C). Average initial sulfolane concentrations in all treatment groups were between 730 μg L^-1^ and 830 μg L^-1^. After nine days of incubation, both live slurry treatments had significantly lower sulfolane concentrations than their sterile counterparts, indicating that biodegradation of sulfolane was occurring (ANOVA, F_3,11_ = 59.88, p<0.001; post-hoc Tukey, p<0.001). Furthermore, hydrocarbon-containing slurries had higher concentrations of sulfolane remaining than the sulfolane-only treatments (p = 0.006). Within 15 days of incubation, sulfolane concentrations in the treatment group without petroleum co-contamination dropped from 730.96 ± 32.85 μg L^-1^ to a non-detectable level in all replicates. In the treatment group containing hydrocarbon co-contamination, sulfolane levels declined to a lesser extent, from an initial sulfolane concentration of 750.48 ± 31.68 μg L^-1^ to 205.25 ± 150.17 μg L^-1^ and was no longer detected after 22 days of incubation. Sulfolane biodegradation rates were calculated to be 48.7 μg L^-1^ day ^-1^ in the non-hydrocarbon containing live slurries and 34.09 μg L^-1^ day ^-1^ in the live slurries containing hydrocarbons. No sulfolane loss was observed in the sterile controls.

#### Dissolved sulfate increases as dissolved sulfolane biodegrades

**Fig 2:**
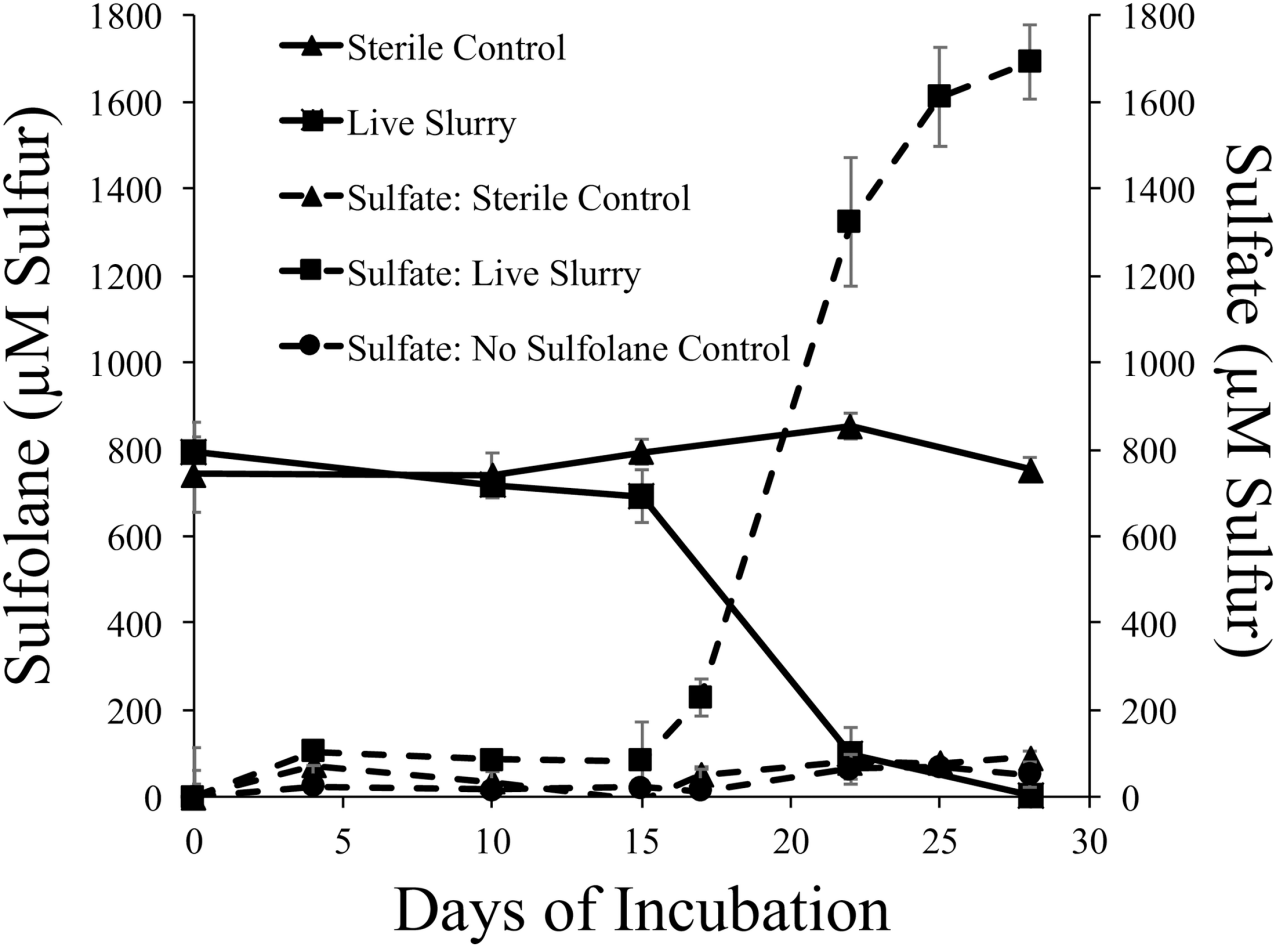
Analysis of dissolved sulfur over time in high concentration, nutrient-amended sediment slurry microcosms. Solid lines indicate dissolved sulfur attributed to sulfolane. Dotted lines indicate dissolved sulfur attributed to sulfate. Sulfate values are normalized to starting concentrations. Error bars indicate standard deviation from the mean.

Dissolved sulfate, a predicted end-product of sulfolane biodegradation, increased in concentration as sulfolane biodegraded (Fig 2). However, much more sulfate was generated than could have originated from sulfolane alone. The concentration of sulfur associated with dissolved sulfate increased from 942.4 μmol L^-1^ to 2634.18 μmol L^-1^ after 28 days of incubation in the nutrient-amended live-treatment group. No significant change in sulfate concentration was observed in either the sterile control or the no-sulfolane control. The sulfur associated with sulfolane in the slurries decreased from a starting concentration of 794.3 μmol L^-1^ to 3.94 μmol L^-1^. Therefore, no more than 790.36 of the 1691.8 μmol sulfur L^-1^ that accumulated in the form of dissolved sulfate can be attributed to sulfolane degradation. Similar trends were observed in the non-nutrient amended incubations.

### Anaerobic incubations

Anaerobic sulfolane biodegradation by aquifer biota was not detected under nitrate, sulfate, or iron reducing conditions (Table 2). Dissolved sulfate and nitrate losses and Fe(II) generation were detected in the benzoate-amended samples indicating the presence of an active anaerobic microbial community. Yet, no sulfolane degradation was observed in any anaerobic microcosm throughout the course of these experiments (1021 days for nitrate and sulfate-reducing conditions and 391 days for iron-reducing conditions).

**Table 2:** Summary of the time required to achieve 95% sulfolane biodegradation for all microcosm studies in aquifer substrate from North Pole, Alaska.

Incubation type	95% Sulfolane degraded (days)
High concentration Slurry	Not achieved: ~ 40% in 106 days
	28*
Low Concentration Slurry	13
	13*
Groundwater Only	No activity
	49*
	80**
Hydrocarbon Co-Contaminated Slurry	22
Anaerobic Sulfate Reducing	No activity
Anaerobic Nitrate Reducing	No activity
Anaerobic Iron Reducing	No activity

* Indicates amendment with a dilute mineral nutrient solution.

** Indicates amendment with an organic nutrient source

## Discussion

### Sulfolane concentrations reduced exclusively via aerobic biodegradation

These microcosm incubation studies demonstrated that aerobic sulfolane biodegradation potential exists in this subarctic aquifer and that biodegradation can occur at *in situ* temperature (4°C) under aerobic conditions. This is consistent with previous reports of aerobic sulfolane biodegradation in aquifer substrate from Western Canada [13,15,18]. However, in contrast to other studies, sulfolane did not biodegrade in the aquifer substrate under anaerobic (nitrate, sulfate, Fe(III)-reducing) conditions. This may be due to biogeographic and/or biogeochemical differences in the microbial communities in this Alaskan aquifer compared to Western Canada. Sulfolane was reported to readily biodegrade in Australian aquifer sediment bioreactors in the absence of oxygen at a temperature of 32°C [17]. Sulfolane also biodegraded in some anaerobic microcosm incubations conducted at 10°C under nitrate- and Mn(IV)- reducing conditions in contaminated aquifer sediment from western Canada [15]. Although sulfolane biodegradation occurred in the Canadian studies, it was not observed in all treatment replicates or at a higher incubation temperature (28°C). Differences in incubation temperatures do not fully account for the inconsistency observed between replicates in the Canadian microcosms. Rather, inconsistent results between treatment replicates suggest there may be an uneven distribution of anaerobic sulfolane degraders in the environment. We incubated samples at 4°C, which is the approximate water temperature of the North Pole subarctic aquifer [22]. Repeating our experiments at higher temperatures might reveal anaerobic sulfolane biodegradation potential if it is present but being limited by temperature.

Although it was not examined in this study, previous research has found sulfolane to anaerobically biodegrade in the presence of Mn (IV) [15]. Since manganese is only sporadically dispersed throughout the aquifer we did not simulate Mn (IV) reducing conditions. Future biodegradation experiments simulating Mn (IV) reducing conditions using subarctic aquifer substrate could reveal if such biodegradation potential exists in the aquifer, elucidate the importance of Mn (IV) on the persistence of sulfolane in this system, and help to reveal the geographic distribution of that trait.

### Mineral nutrients stimulate biodegradation rates at high concentrations; no effect at low

Mineral nutrient amendment of sediment slurry microcosms stimulated aerobic biodegradation at high sulfolane concentrations (100 mg L^-1^), but not at low sulfolane concentrations (500 μg L^-1^) (Fig 1A and 1B). This difference is likely related to the difference in nutrient requirements necessary to process differing amounts of a substrate. Our studies are the first part-per-billion biodegradation assays on aquifer substrate attempting to mimic subarctic aquifer conditions. We report an average biodegradation rate of 38.96 μg L ^-1^ day ^-1^ in the low concentration sediment slurries regardless of nutrient amendments. This suggests that the North Pole aquifer has sufficient ambient nutrients to support microbial processing of small quantities of sulfolane. However, in an aquifer the movement of groundwater tends to replenish contaminants at a given location. Therefore, it is unknown whether nutrient additions *in situ* would be necessary to maintain sulfolane biodegradation within the aquifer, given sufficient oxygen.

At high experimental sulfolane concentrations (100 mg L^-1^) we found that amendment with a mineral nutrient solution increased the aerobic biodegradation rate from 2.93 mg L^-1^ day^-1^ to 6.19 mg L^-1^ day^-1^ and promoted complete sulfolane removal (Fig 1A). This is consistent with previous biodegradation findings in sediment and groundwater from a contaminated aquifer in Western Canada [13,18]. In aerobic shake flask microcosms containing 20 mg L^-1^ sulfolane and incubated at 8°C, biodegradation rates increased from 0.8–1 mg L^-1^ day^-1^ to 4 mg L^-1^ day^-1^ after the addition of N and P [13]. Another study from Western Canada using contaminated soil as the only inoculum found that N and P addition reduced the lag time in sulfolane biodegradation activity from 77 days to 2 days and increased biodegradation rates from 4.56 mg L^-1^ day^-1^ to 45.6 mg L^-1^ day^-1^ [18]. Observing similar results from different environmental samples suggests that nutrient limitation may be a universal constraint on aerobic sulfolane biodegradation at high sulfolane concentrations.

We also observed slightly higher degradation rates than Fedorak and Coy (1996) despite having a lower incubation temperature (4°C vs. 8°C). This was unexpected since temperature has been positively correlated with sulfolane biodegradation activity [13,15] and enzymatic activity in general [27,28]. The discrepancy may be due to differences in the amount of sediment used as the source of inoculum [29,30]. Fedorak and Coy (1996) used 50 g of aquifer sediment and 450 ml of groundwater (1:9 sediment water ratio) while we used 25 g of sediment and 100 ml of groundwater (1:4 ratio). Alternatively, differences between biodegradation rates might be accounted for by community composition differences between samples. Plate counts have shown that the abundance of sulfolane degraders is variable between samples [16,18]. Further investigations identified a *Variovorax sp*. as being capable of mineralizing sulfolane, although mixed cultures demonstrated greater mineralization than isolated degraders [19]. Although modern molecular techniques such as Next Gen sequencing have not yet been employed on this topic, determining the identity of sulfolane degraders and their *in situ* distribution in respect to environmental variables may reveal other controls on sulfolane biodegradation; enabling more accurate estimates of plume longevity. Therefore, future work should be focused on examining the microbial community involved in active sulfolane biodegradation and determining the spatial distribution of specific sulfolane degraders.

### Nutrient addition necessary for biodegradation in groundwater alone

The water table is close to the ground surface in North Pole, AK and often needs to be lowered during the construction season for building activities to occur. Water is pumped out of the ground and transported elsewhere through a process known as dewatering [31]. Typically, extracted groundwater is discharged to the ground surface in drainage ditches that connect to established stormwater flow systems (Alaska Department of Environmental Conservation, personal communication). If the groundwater contains sulfolane, this process could increase human exposure risk and contaminate previously uncontaminated areas. Therefore, examining the biodegradation potential of the planktonic community in groundwater has implications for dewatering waste management strategies. Since the majority of the microbial biomass in aquifers is thought to be associated with the aquifer sediment [21], we predicted that the biodegradation potential of sulfolane in groundwater alone would be lower than that of sediment slurries.

No sulfolane biodegradation occurred in groundwater without the addition of nutrients, indicating that sulfolane biodegradation is limited at *in situ* sulfolane concentrations (Fig 1C). The impact of nutrient amendment on groundwater-only microcosms was unexpected, as there was no difference in biodegradation rates between nutrient amended and non-nutrient amended microcosms containing sediment at similar sulfolane concentrations. This difference might be explained by the differences in biomass and/or microbial community composition between sediment and groundwater [21,32]. It is also possible that this difference is due to the presence of nutrients in aquifer sediment, which may be sufficient to support active microbial growth while those available in groundwater alone are too limited. Mineral nutrients stimulated sulfolane biodegradation in groundwater more effectively than complex organic nutrients (beer fermentation settlings), but both nutrient additions were effective at increasing the biodegradation rate. This may be related to the fact that mineral nutrients are more bioavailable than complex organics; ammonium being the preferred nitrogen source for bacteria [33]. Labile organic carbon might also have been preferentially utilized over the more recalcitrant sulfolane, slowing degradation rates [34].

### Hydrocarbon co-contamination retards sulfolane biodegradation in alluvial substrate

Petroleum hydrocarbon contamination has also been found in the groundwater on the refinery; mainly in the form of jet and diesel fuel [22]. We demonstrated that kerosene, which is similar in composition to onsite hydrocarbon contaminants, retards the rate of sulfolane biodegradation in aerobic sediment slurries by approximately 30% (Fig 1C). This finding agrees with previous research that found sulfolane biodegradation rates to be lower than those for common co-contaminants, such as diisopropylamine and hydrocarbons [6,7] although to our knowledge no competitive degradation experiments have previously been conducted. Our findings suggest co-contaminants are utilized preferentially over sulfolane and/or there is a toxic effect of co-contaminants on sulfolane-degrading microorganisms. Therefore, the suppressive effects of hydrocarbons on sulfolane biodegradation rates should be taken into consideration when modeling sulfolane biodegradation in co-contaminated aquifers.

### Mineralization product of sulfolane biodegradation produced

The end products of sulfolane mineralization are proposed to be carbon dioxide and sulfate [19]. Previous biodegradation experiments found that up to 97% of the sulfur in sulfolane was converted into sulfate in mixed culture incubations, suggesting that complete mineralization of sulfolane was occurring. We also found that sulfate was produced while sulfolane biodegraded in our microcosm studies and similarly sulfate was the only biodegradation product we detected. However, much more sulfate was detected than could have originated from sulfolane alone (Fig 2). This discrepancy may be due to the additional degradation of other organosulfur-compounds in the sediment, which contain functional groups such as sulfonates and sulfate esters [35–37]. It is also possible that microorganisms are liberating sulfur from sulfur containing minerals (e.g. pyrite) as a result of biological activity [38,39]. However, biological activity alone cannot account for this discrepancy, as there was no sulfate produced in the sulfolane-free live slurry, which also contained live microorganisms. It is well known that supplying a microbial community with an abundance of a specific substrate can stimulate the growth of organisms capable of utilizing the substrate and similar compounds while suppressing the growth of those that cannot (e.g. [40]). We propose that a similar situation is occurring in our microcosm studies and that an amendment with the organosulfur molecule, sulfolane, stimulates the growth of microorganisms able to degrade many types of organosulfur molecules naturally occurring in aquifer sediment. In an aerobic aqueous solution, the end product of the sulfur atom removed from organosulfur compounds during biodegradation is often sulfate [36].

Our results suggest that an increase in sulfate concentrations observed in complex media is indicative of sulfolane biodegradation, yet represents a combination of sulfate liberated from sulfolane and other sulfur compounds found in the aquifer materials. Therefore, to conclusively determine if biodegradation has occurred, sulfolane concentrations should always be measured, rather than using sulfate production alone as a proxy. The degradation pathway for sulfolane has not yet been elucidated and it is not known what, if any, biodegradation intermediates accumulate. Isotopic analyses of sulfolane and its degradation products containing isotopically labeled sulfur could also be fruitful for identifying the pathways involved.

We found that sulfolane biodegraded only in the presence of oxygen. We also demonstrated that ambient nutrient concentrations were sufficient for sulfolane biodegradation to occur at the sulfolane concentrations being detected within the plume, but only when oxygen is available. The results of our research suggest that the sulfolane contamination associated with the Tanana aquifer in North Pole, Alaska, is not likely to undergo biodegradation under ambient aquifer conditions, with possible exceptions being locations where trace levels of oxygen may be present, such as the leading edge of the contaminant plume, locations where groundwater and surface waters interact (e.g., edges of surface water bodies, such as ponds), or possibly in shallow portions of the aquifer susceptible to infiltration (e.g., oxygenated stormwater runoff). It has been shown that the leading edge of contaminant plumes tend to have dissolved oxygen that gets consumed as organic contaminants degrade; a phenomenon known as the plume “fringe” effect [41]. Groundwater monitoring wells along the North Pole, Alaska sulfolane plume fringe have dissolved oxygen concentrations up to 5 mg L^-1^ [22]. Our studies were only conducted under fully aerated conditions so it remains uncertain whether sulfolane biodegradation can occur under the low-oxygen conditions observed *in situ*, including at plume fringes. A similar effect may be observed at the groundwater-surface water interface of gravel ponds. Determining if sulfolane biodegradation occurs under suboxic conditions characteristic of those at the plume “fringe” or other oxygenated regions of the plume would allow for more accurate estimates of the contaminant’s fate and transport. Also techniques such as “air sparging”, where the aquifer material is flushed with atmospheric air to stimulate sulfolane biodegradation *in situ* should be further researched as a localized remediation strategy for sulfolane contaminated aquifer substrate [18,42].

## Conclusion

The subarctic aquifer that underlies North Pole, Alaska contains an active microbial community capable of performing aerobic sulfolane biodegradation, however oxygen is likely the primary limiting factor *in situ*. The presence of petroleum co-contamination retards aerobic sulfolane biodegradation, and may contribute to low degradation rates in the subsurface. At the sulfolane concentrations prevalent in the plume, nutrient levels were sufficient to support biodegradation when sufficient oxygen was present, so nutrient addition would not be expected to accelerate biodegradation in the plume. Our study reinforces the importance for researchers modeling sulfolane half-lives under various aquifer conditions to not only incorporate a biodegradation term in their models, but also to consider the variability of biodegradation rates associated with differing environmental conditions, including oxygen availability and co-contamination. The microbial community associated with groundwater alone has a lower biodegradation potential than that associated with groundwater-sediment mixtures, however nutrient amendments were successful in stimulating aerobic degradation in groundwater alone, which has implications for remediation of dewatering waste. Anaerobic conditions do not appear to support sulfolane biodegradation. Yet low oxygen conditions, such as those that often prevail at the leading edge of a plume, may have the potential to foster biodegradation activity as seen for some other organic contaminants [41], but warrants further investigation.

Future work should be directed at elucidating the identity of the microorganisms involved in sulfolane biodegradation. Doing so may reveal new taxa as well as provide taxonomic indicators of the potential for active sulfolane biodegradation *in situ* at a contaminated site. Determining the distribution and potential activity of sulfolane-degrading microorganisms under the range of redox and biogeochemical conditions present, including suboxic conditions, would also aid efforts to more accurately predict the fate of sulfolane in the environment and to perform monitored natural attenuation.

## Supporting information

S1 File Sulfolane data for microcosm studies. (XLSX)
